# Supplementary material for: Lying in Wait: Modeling the Control of Bacterial Infections via Antibiotic-Induced Proviruses
Source: mSystems. 2019 Oct 1;4(5):e00221-19. doi: 10.1128/mSystems.00221-19 (PMC6774016; doi:10.1128/mSystems.00221-19)
Supplement: TEXT S1 [file mSystems.00221-19-s0001.pdf]

MODEL

$$\frac{\mathrm{d}S}{\mathrm{d}t} = \underbrace{\gamma S \left(1 - \frac{B_{\mathrm{tot}}}{K}\right)}_{\text{growth}} - \underbrace{\eta\, r(V_{\mathrm{tot}}, S)}_{\text{infection}} - \underbrace{\kappa\, s(t, \{t_i\}) S}_{\text{antibiotic}} \tag{S1}$$

$$\frac{\mathrm{d}I_T}{\mathrm{d}t} = \underbrace{\eta(1 - f_T)\, r(V_T, S)}_{\text{infection}} - \underbrace{\delta I_T}_{\text{lysis}} + \underbrace{s(t, \{t_i\})(L_T + L_{CT}^{(T)} + P_{CT}^{(T)})}_{\text{induced lysis}} - \underbrace{\kappa\, s(t, \{t_i\}) I_T}_{\text{antibiotic}} \tag{S2}$$

$$\frac{\mathrm{d}I_C}{\mathrm{d}t} = \underbrace{\eta(1 - f_C)\, r(V_C, S)}_{\text{infection}} - \underbrace{\delta I_C}_{\substack{\text{become}\\\text{productive}}} - \underbrace{\kappa\, s(t, \{t_i\}) I_C}_{\text{antibiotic}} \tag{S3}$$

$$\frac{\mathrm{d}L_T}{\mathrm{d}t} = \underbrace{\gamma L_T \left(1 - \frac{B_{\mathrm{tot}}}{K}\right)}_{\text{growth}} + \underbrace{\eta f_T\, r(V_T, S)}_{\substack{\text{latent}\\\text{infection}}} - \underbrace{\eta\, r(V_C, L_T)}_{\substack{\text{cross}\\\text{infection}}} - \underbrace{s(t, \{t_i\}) L_T}_{\text{induced lysis}} - \underbrace{\kappa\, s(t, \{t_i\}) L_T}_{\text{antibiotic}} \tag{S4}$$

$$\frac{\mathrm{d}P_C}{\mathrm{d}t} = \underbrace{\lambda\gamma\, g\big(s(t, \{t_i\})\big) P_C \left(1 - \frac{B_{\mathrm{tot}}}{K}\right)}_{\text{growth}} + \underbrace{\delta I_C}_{\substack{\text{become}\\\text{productive}}} - \underbrace{\eta\, r(V_T, P_C)}_{\substack{\text{cross}\\\text{infection}}} - \underbrace{\kappa\, s(t, \{t_i\}) P_C}_{\text{antibiotic}} \tag{S5}$$

$$\frac{\mathrm{d}L_C}{\mathrm{d}t} = \underbrace{\gamma L_C \left(1 - \frac{B_{\mathrm{tot}}}{K}\right)}_{\text{growth}} + \underbrace{\eta f_C\, r(V_C, S)}_{\substack{\text{latent}\\\text{infection}}} - \underbrace{\eta\, r(V_T, L_C)}_{\substack{\text{cross}\\\text{infection}}} - \underbrace{\kappa\, s(t, \{t_i\}) L_C}_{\text{antibiotic}} \tag{S6}$$

$$\frac{\mathrm{d}I_{CT}^{(T)}}{\mathrm{d}t} = \underbrace{\eta(1 - f_C)\, r(V_C, L_T)}_{\substack{\text{cross}\\\text{infection}}} - \underbrace{\delta I_{CT}^{(T)}}_{\substack{\text{become}\\\text{productive}}} - \underbrace{\kappa\, s(t, \{t_i\}) I_{CT}^{(T)}}_{\text{antibiotic}} \tag{S7}$$

$$\frac{\mathrm{d}I_{CT}^{(P)}}{\mathrm{d}t} = \underbrace{\eta(1 - f_T)\, r(V_T, P_C)}_{\substack{\text{cross}\\\text{infection}}} - \underbrace{\delta I_{CT}^{(P)}}_{\text{lysis}} + \underbrace{s(t, \{t_i\}) P_{CT}^{(C)}}_{\text{induced lysis}} - \underbrace{\kappa\, s(t, \{t_i\}) I_{CT}^{(P)}}_{\text{antibiotic}} \tag{S8}$$

$$\frac{\mathrm{d}I_{CT}^{(C)}}{\mathrm{d}t} = \underbrace{\eta(1 - f_T)\, r(V_T, L_C)}_{\substack{\text{cross}\\\text{infection}}} - \underbrace{\delta I_{CT}^{(C)}}_{\text{lysis}} + \underbrace{s(t, \{t_i\}) L_{CT}^{(C)}}_{\text{induced lysis}} - \underbrace{\kappa\, s(t, \{t_i\}) I_{CT}^{(C)}}_{\text{antibiotic}} \tag{S9}$$

$$\frac{\mathrm{d}L_{CT}^{(T)}}{\mathrm{d}t} = \underbrace{\gamma L_{CT}^{(T)} \left(1 - \frac{B_{\mathrm{tot}}}{K}\right)}_{\text{growth}} + \underbrace{\eta f_C\, r(V_C, L_T)}_{\substack{\text{latent}\\\text{infection}}} - \underbrace{s(t, \{t_i\}) L_{CT}^{(T)}}_{\text{induced lysis}} - \underbrace{\kappa\, s(t, \{t_i\}) L_{CT}^{(T)}}_{\text{antibiotic}} \tag{S10}$$

$$\frac{\mathrm{d}P_{CT}^{(T)}}{\mathrm{d}t} = \underbrace{\lambda\gamma g\big(s(t, \{t_i\})\big) P_{CT}^{(T)} \left(1 - \frac{B_{\mathrm{tot}}}{K}\right)}_{\text{growth}} + \underbrace{\delta I_{CT}^{(T)}}_{\substack{\text{become}\\\text{productive}}} - \underbrace{s(t, \{t_i\}) P_{CT}^{(T)}}_{\text{induced lysis}} - \underbrace{\kappa\, s(t, \{t_i\}) P_{CT}^{(T)}}_{\text{antibiotic}} \tag{S11}$$

$$\frac{\mathrm{d}L_{CT}^{(C)}}{\mathrm{d}t} = \underbrace{\gamma L_{CT}^{(C)} \left(1 - \frac{B_{\mathrm{tot}}}{K}\right)}_{\text{growth}} + \underbrace{\eta f_T\, r(V_T, L_C)}_{\substack{\text{latent}\\\text{infection}}} - \underbrace{s(t, \{t_i\}) L_{CT}^{(C)}}_{\text{induced lysis}} - \underbrace{\kappa\, s(t, \{t_i\}) L_{CT}^{(C)}}_{\text{antibiotic}} \tag{S12}$$

$$\frac{\mathrm{d}P_{CT}^{(C)}}{\mathrm{d}t} = \underbrace{\lambda\gamma\, g\big(s(t, \{t_i\})\big) P_{CT}^{(C)} \left(1 - \frac{B_{\mathrm{tot}}}{K}\right)}_{\text{growth}} + \underbrace{\eta f_T\, r(V_T, P_C)}_{\substack{\text{latent}\\\text{infection}}} - \underbrace{s(t, \{t_i\}) P_{CT}^{(C)}}_{\text{induced lysis}} - \underbrace{\kappa\, s(t, \{t_i\}) P_{CT}^{(C)}}_{\text{antibiotic}} \tag{S13}$$

$$\frac{\mathrm{d}V_T}{\mathrm{d}t} = \underbrace{\beta_T \delta \left(I_T + I_{CT}^{(P)} + I_{CT}^{(C)}\right)}_{\text{bursting}} - \underbrace{\eta\, r(V_T, S + P_C + L_C)}_{\text{adsorption}} - \underbrace{d V_T}_{\text{degradation}} \tag{S14}$$

$$\frac{\mathrm{d}V_C}{\mathrm{d}t} = \underbrace{b\big(s(t, \{t_i\})\big) \left(P_C + P_{CT}^{(T)} + P_{CT}^{(C)}\right)}_{\text{phage production}} - \underbrace{\eta\, r(V_C, S + L_T)}_{\text{adsorption}} - \underbrace{d V_C}_{\text{degradation}} \tag{S15}$$
