# Supplementary material for: Lying in Wait: Modeling the Control of Bacterial Infections via Antibiotic-Induced Proviruses
Source: mSystems. 2019 Oct 1;4(5):e00221-19. doi: 10.1128/mSystems.00221-19 (PMC6774016; doi:10.1128/mSystems.00221-19)
Supplement: TEXT S2 [file mSystems.00221-19-s0002.pdf]

PARAMETER SELECTION: TECHNICAL DETAILS

Model non-dimensionalization

The model (S1-S15) can be non-dimensionalized so that time and density are unitless quantities. We will illustrate with (S1), but the methodology is analogous for all other equations in the system. Suppose the system (S1-S15) has bacterial density units of CFU/mL and time units of minutes. Then equation (S1) has units CFU/mL/min:

$$\frac{\mathrm{d}S}{\mathrm{d}t} = \underbrace{\gamma S\left(1-\frac{B_{\text{tot}}}{K}\right)}_{\text{growth}} - \underbrace{\eta\, V_{\text{tot}}\frac{S}{h_\eta + S}}_{\text{infection}} - \underbrace{\kappa\, s(t,\{t_i\})S}_{\text{antibiotic}}.$$

We will now divide the entire equation by γK, i.e. the bacterial growth rate (in min^{-1}) multiplied by the carrying capacity (in CFU/mL):

$$\frac{\mathrm{d}(S/K)}{\mathrm{d}(\gamma t)} = \underbrace{\frac{S}{K}\left(1-\frac{B_{\text{tot}}}{K}\right)}_{\text{growth}} - \underbrace{\frac{\eta}{\gamma}\,\frac{V_{\text{tot}}}{K}\,\frac{S/K}{h_\eta/K + S/K}}_{\text{infection}} - \underbrace{\kappa\,\frac{s(t,\{t_i\})}{\gamma}\,\frac{S}{K}}_{\text{antibiotic}}.$$

Now $\tilde{S} = S/K$ is a unitless bacterial density, $\tilde{t} = \gamma t$ is a unitless time, $\tilde{\eta} = \eta/\gamma$ is a unitless infection rate, and $\tilde{V}_{\text{tot}} = V_{\text{tot}}/K$ is a unitless phage to bacteria ratio (if the bacterial population is at carrying capacity):

$$\frac{\mathrm{d}\tilde{S}}{\mathrm{d}\tilde{t}} = \underbrace{\tilde{S}\left(1-\tilde{B}_{\text{tot}}\right)}_{\text{growth}} - \underbrace{\tilde{\eta}\,\tilde{V}_{\text{tot}}\frac{\tilde{S}}{\tilde{h}_\eta + \tilde{S}}}_{\text{infection}} - \underbrace{\kappa\,\tilde{s}(\tilde{t},\{\tilde{t}_i\})\tilde{S}}_{\text{antibiotic}}.$$

For the sake of clarity, we have suppressed tilde notation throughout the main manuscript. The same effect is achieved by choosing $\gamma = 1$ and $K = 1$ in system (S1-S15) and reinterpreting bacterial populations as fractions of the carrying capacity and rates as multiples of the growth rate. All parameters in Table II have been scaled as such (details follow).

Parameter non-dimensionalization

The **growth rate** γ for *P. aeruginosa in vitro* is approximately 5.1e-3 min^{-1} [1], although *P. aeruginosa* growth is highly variable in patients with cystic fibrosis [2]. Therefore all rate parameters provided in min^{-1} are scaled by this rate in order to non-dimensionalize.

The **carrying capacity** K of bacteria in sputum is difficult to estimate due to variability within patients. One study of patients with cystic fibrosis found that the densities of viable *P. aeruginosa* in sputum of 12 patients not undergoing treatment ranged from 5.3e3 CFU/mL to 1.8e11 CFU/mL [3]. Let us say that a 'typical' carrying capacity is the geometric mean of this range: 3.1e7 CFU/mL. For reasons that will be explained in the following paragraphs, we round up to K =4e7 CFU/mL. Therefore we scale all bacterial densities provided in CFU/mL by this density in order to non-dimensionalize.

The **infection rate** for *E. coli* and T4 phage in mucus (assuming mass action infection) is known to be approximately 47e-10 mL/min [4, 5]. Infection in marine ecosystems (also assuming mass action infection) is similar at around 24e-10 mL/min [4, 6]. Because our model assumes Holling's type II infection rather than mass action (i.e., Holling's type I) infection, scaling the infection rate is not straightforward. If the mass action infection rates η_{ma} have been estimated in low population density conditions (i.e., $B_{\mathrm{tot}} << h_\eta$), then $\eta_{\mathrm{ma}} \approx \frac{\eta}{h_\eta}$. If instead the bacterial population is large (i.e., $B_{\mathrm{tot}} >> h_\eta$), then $\eta_{\mathrm{ma}} \approx \eta$. Most likely, the infection rates were not estimated in either of these extreme conditions, and therefore η will fall somewhere between η_{ma} and $h_\eta \eta_{\mathrm{ma}}$.

Given that we do not know h_η, we elected to use the non-dimensional η given by Sinha et al. [7], assuming that the population is large; this is reasonable because the authors fit their mass action infection model to time series population data that reached carrying capacity. In all demonstrations, the authors selected a non-dimensional $\eta = 20$, which we also use. On the way to selecting that value, the authors presented $K\eta \in [0.45, 100]$ hr^{-1} and $\gamma \in [0.5, 10]$ hr^{-1}. Non-dimensionalization leads to a range of η between 0.045 and 200, and the selected value of 20 falls in that range.

We have arbitrarily selected $h_\eta = K/2$, implying that the bacterial population for which the infection rate is half the maximum is half the carrying capacity. We will now verify that this selection is reasonable even if the population is small (i.e., $B_{\mathrm{tot}} << h_\eta$). As we stated earlier, it is known that the mass action infection rate for *E. coli* and T4 phage in mucus is approximately 47e-10 mL/min [4, 5]. Assuming small bacterial populations, the following statement should hold: $47\mathrm{e}{-10} \approx \frac{20\times 5.1\mathrm{e}{-3}}{h_\eta}$. Solving for h_η gives $h_\eta \approx$ 2e7 CFU/mL. This is half the carrying capacity K that we selected for scaling all density parameters.

The **amplitude of stress** A is estimated using the rate at which meropenem in combination with antimicrobial peptides induces morphological changes in *P. aeruginosa* [1]. The morphological transition rate is given as 5.4e-3 min^{-1}; non-dimensionalizing by the growth rate γ = 5.1e-3, we get $A \approx 1.1$.

The **metabolic decay rate of antibiotics** k is calculated based on the half-life of Levofloxacin [8]. The half-life of a standard dose of Levofloxacin within a human is approximately $T_{1/2} = 6.9$ hours = 414 minutes; the metabolic decay rate is then $k = \ln(2)/T_{1/2} \approx 1.7\text{e-}3 \text{ min}^{-1}$; non-dimensionalizing by the growth rate $\gamma = 5.1\text{e-}3$, we get $k \approx 0.3$.

The **phage production delay rate** δ is estimated based on the eclipse and rise phase of PAXYB1 and PAK_P3 phage [9, 10]. The eclipse (latent) and rise phase is 130 minutes total for PAXYB1 [9] and 27 minutes total for PAK_P3 [10]. The smallest (non-dimensional) delay rate is then 1/130/5.1e-3= 1.5, and the largest is 1/27/5.1e-3= 7.3. We selected the approximate average of this range, 4, to be the delay rate.

The values of h_β and h_γ were selected to be 1 for simplicity. This means the stress level for which the production rate is halfway between the minimum and maximum is 1 after non-dimensionalization. Similarly, the stress level for which the growth rate is half the maximum is 1 after non-dimensionalization. The values of h_β and h_γ are both in the same units of stress (min^{-1}). In those units, $h_\beta = h_\gamma = 5.1\text{e-}3 \text{ min}^{-1}$.

SENSITIVITY ANALYSIS: TECHNICAL DETAILS

In all analyses, we have used the initial conditions $S(0) = 1\text{e}{-3}, V_T(0) = V_C(0) = 1\text{e}{-7}$, following Sinha et al. [7], unless otherwise noted. While the steady state dynamics are not identical under different initial conditions, we note that the qualitative steady state behaviors (e.g., surviving strain types, dominant phage strategies, peak bacterial population sizes during antibiotic treatment, etc.) are robust to initial condition changes. Also, we have demonstrated in Fig 7 that the initial ratio of phage types does not significantly impact either the minimum antibiotic dosing period or the minimum deadliness needed to control the infection.

The global uncertainty and sensitivity analysis was performed using the methodology outlined in Marino et al. [11]. The base code for the analysis is freely available at the author Denise Kirschner's website [12]. The specific implementation is available at the Illinois Data Bank repository: `doi.org/10.13012/B2IDB-9721455_V1` [13].

In brief, the analysis uses Latin Hypercube Sampling (LHS) of parameter space to simulate uncertainty in model parameters. LHS sampling requires fewer model simulations than simple random sampling without introducing bias [14]. We used uniform sampling of each parameter about the base values given in Table II. The ranges of the uniform samples are available in our

code.

We use Partial Rank Correlation Coefficients (PRCC) to test the sensitivity of model outputs to parameter uncertainty because model outputs generally depend monotonically on model inputs, but those relationships are not linear trends. As noted by Marino et al. [11], for linear trends we could have used Pearson correlation coefficient (CC), partial correlation coefficients (PCCs), or standardized regression coefficients (SRC). Had our trends been non-monotonic, we would have used the Sobol method or one of its many extensions [15].

The displayed sensitivities in Fig 7 are the PRCCs for the model output y (either minimum T or minimum κ) and the model inputs x_j. As described by Marino et al. [11], partial rank correlation characterizes the monotonic relationship between input x_j and output y after the effects on y of the other inputs are removed. The values of PRCCs fall between -1 and 1, with 1 indicating the strongest positive rank correlation and -1 indicating the strongest negative rank correlation. The significance indicates the probability that the rank correlation is zero (i.e., large significance suggests that there is no relationship between x_j and y).

---

[1] C. Spalding, E. Keen, D. J. Smith, A.-M. Krachler, and S. Jabbari, "Mathematical modelling of the antibiotic-induced morphological transition of pseudomonas aeruginosa," *PLoS computational biology*, vol. 14, no. 2, p. e1006012, 2018.

[2] S. H. Kopf, A. L. Sessions, E. S. Cowley, C. Reyes, L. Van Sambeek, Y. Hu, V. J. Orphan, R. Kato, and D. K. Newman, "Trace incorporation of heavy water reveals slow and heterogeneous pathogen growth rates in cystic fibrosis sputum," *Proceedings of the National Academy of Sciences*, vol. 113, no. 2, pp. E110–E116, 2016.

[3] F. A. Stressmann, G. B. Rogers, P. Marsh, A. K. Lilley, T. W. Daniels, M. P. Carroll, L. R. Hoffman, G. Jones, C. E. Allen, N. Patel, N. Forbes, B. Forbes, A. Tuck, and K. D. Bruce, "Does bacterial density in cystic fibrosis sputum increase prior to pulmonary exacerbation?," *Journal of Cystic Fibrosis*, vol. 10, no. 5, pp. 357–365, 2011.

[4] G. S. Stent *et al.*, "Molecular biology of bacterial viruses.," *Molecular biology of bacterial viruses.*, 1963.

[5] J. J. Barr, R. Auro, N. Sam-Soon, S. Kassegne, G. Peters, N. Bonilla, M. Hatay, S. Mourtada, B. Bailey, M. Youle, *et al.*, "Subdiffusive motion of bacteriophage in mucosal surfaces increases the frequency of bacterial encounters," *Proceedings of the National Academy of Sciences*, vol. 112, no. 44, pp. 13675–13680, 2015.

[6] T. F. Thingstad, S. Våge, J. E. Storesund, R.-A. Sandaa, and J. Giske, "A theoretical analysis of how strain-specific viruses can control microbial species diversity," *Proceedings of the National Academy of*

Sciences, vol. 111, no. 21, pp. 7813–7818, 2014.

[7] V. Sinha, A. Goyal, S. L. Svenningsen, S. Semsey, and S. Krishna, "In silico evolution of lysis-lysogeny strategies reproduces observed lysogeny propensities in temperate bacteriophages," *Frontiers in microbiology*, vol. 8, p. 1386, 2017.

[8] G. G. Zhanel, S. Fontaine, H. Adam, K. Schurek, M. Mayer, A. M. Noreddin, A. S. Gin, E. Rubinstein, and D. J. Hoban, "A review of new fluoroquinolones," *Treatments in respiratory medicine*, vol. 5, no. 6, pp. 437–465, 2006.

[9] X. Yu, Y. Xu, Y. Gu, Y. Zhu, and X. Liu, "Characterization and genomic study of "phikmv-like" phage paxyb1 infecting pseudomonas aeruginosa," *Scientific reports*, vol. 7, no. 1, p. 13068, 2017.

[10] G. El Didamony, A. Askora, and A. A. Shehata, "Isolation and characterization of t7-like lytic bacteriophages infecting multidrug resistant pseudomonas aeruginosa isolated from egypt," *Current microbiology*, vol. 70, no. 6, pp. 786–791, 2015.

[11] S. Marino, I. B. Hogue, C. J. Ray, and D. E. Kirschner, "A methodology for performing global uncertainty and sensitivity analysis in systems biology," *Journal of theoretical biology*, vol. 254, no. 1, pp. 178–196, 2008.

[12] "Uncertainty and sensitivity functions and implementation." `http://malthus.micro.med.umich.edu/lab/usadata/`. Accessed: 2019-06-22.

[13] Z. Rapti, "Control of bacterial infections via antibiotic-induced proviruses," 2019.

[14] M. D. McKay, R. J. Beckman, and W. J. Conover, "Comparison of three methods for selecting values of input variables in the analysis of output from a computer code," *Technometrics*, vol. 21, no. 2, pp. 239–245, 1979.

[15] A. Saltelli, "Making best use of model evaluations to compute sensitivity indices," *Computer physics communications*, vol. 145, no. 2, pp. 280–297, 2002.
