# Supplementary material for: Lying in Wait: Modeling the Control of Bacterial Infections via Antibiotic-Induced Proviruses
Source: mSystems. 2019 Oct 1;4(5):e00221-19. doi: 10.1128/mSystems.00221-19 (PMC6774016; doi:10.1128/mSystems.00221-19)
Supplement: FIG S1 [file mSystems.00221-19-sf001.pdf]

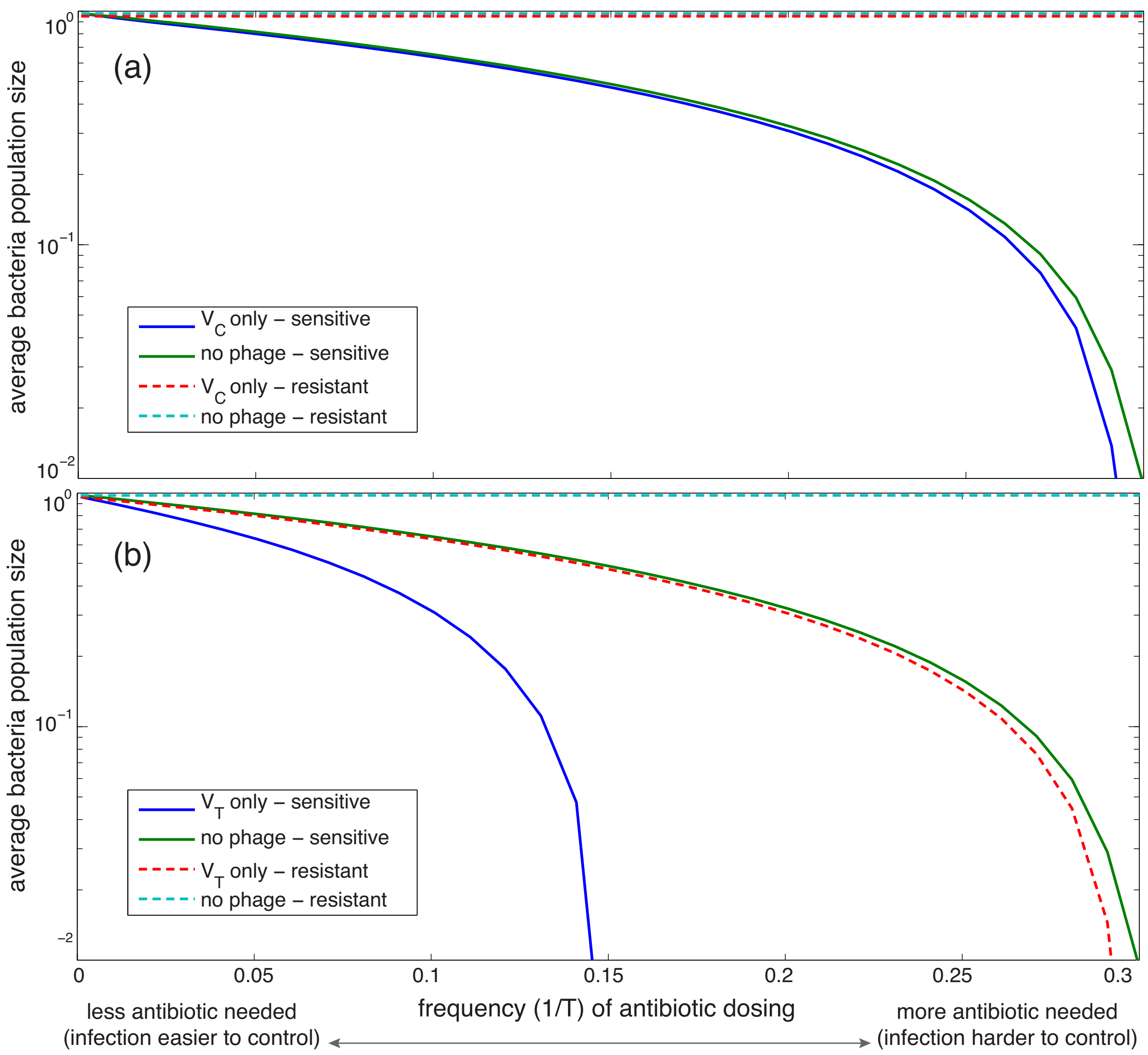

FIG. S1. Average total bacterial population for a range of periodic antibiotic dosing protocols and only one type of phage. (a) No temperate phage present in system. (b) No chronic phage present in system. All parameter values are taken at the baselines in Table II, with $h_\eta = 1/2, h_\beta = 1, h_\gamma = 1, t_{\max} = 300$. Solid lines indicate that all bacteria are sensitive to antibiotics, and dashed lines indicate that all bacteria are resistant. Initially, $S(0) = 1\text{e}{-3}, V_T(0) = V_C(0) = 1\text{e}{-7}$, unless otherwise noted.
